# Environmental and human health impact of contact precaution use for methicillin-resistant *Staphylococcus aureus* and vancomycin-resistant *Enterococcus* in Los Angeles County

**DOI:** 10.1017/ice.2025.10338

**Published:** 2026-01

**Authors:** Pamela S Lee, Kelsey OYong, Ami N. Shah, Cassandra Thiel, Michelle LeBrun, Loren G. Miller, Zachary Rubin

**Affiliations:** 1 Department of Medicine, Division of Infectious Diseases, Harbor-UCLA-Medical Center, Torrance, CA, USA; 2 Division of Infectious Diseases, The Lundquist Institute for Biomedical Innovation, Torrance, CA, USA; 3 Department of Medicine, David Geffen School of Medicine at UCLA, Los Angeles, CA, USA; 4 Los Angeles County Department of Public Health, Los Angeles, CA, USA; 5 Division of Pediatric Surgery, Rush University Medical Center, Chicago, IL, USA; 6 Departments of Population Health and Ophthalmology, NYU Langone Health, New York, NY, USA; 7 Clinically Sustainable Consulting, Madison, WI, USA; 8 Division of Infectious Diseases, Rancho Los Amigos National Rehabilitation Center, Los Angeles, CA, USA

## Abstract

In LA County, contact precautions for methicillin-resistant *Staphylococcus aureus* and vancomycin-resistant *Enterococcus* require 7.3 million gowns annually generating 506 tons of plastic waste and 1.73 million kilograms of carbon dioxide equivalents, which cause the loss of 4.07 disability-adjusted life-years. Unintended consequences of gown use necessitates exploration of infection prevention alternatives.

## Introduction

Production and use of single-use plastics in healthcare continue to escalate, despite poor health outcomes linked to these materials.^1,2^ US hospitals produce >5.9 million tons of waste annually, including 1.7 million tons of plastic waste.^3^ Disposable personal protective equipment (PPE) comprises up to 60% of inpatient plastic waste.^4^ Infection prevention and control practices, such as the use of certain PPE, create significant waste as a byproduct while pursuing the goal of patient safety—waste that may have a devastating health impact.

Contact precautions are recommended for patients infected or colonized with multi-drug resistant organisms (MDROs) such as methicillin-resistant *Staphylococcus aureus* (MRSA) and vancomycin-resistant *Enterococcus* (VRE) to reduce spread of these MDROs in healthcare settings.^5^ However, contact precautions may not reliably prevent endemic MRSA/VRE transmission.^6^ Moreover, contact precaution use has been associated with fewer healthcare worker visits, patient psychological harm, and increased hospital costs,^6^ while discontinuing MRSA/VRE contact precautions has been linked to declines in noninfectious adverse events without increases in MRSA/VRE infections.^7,8^ Many hospitals have thus discontinued routine MRSA/VRE contact precautions,^6^ but their use remains prevalent.

Most contact precautions utilize single-use disposable plastic PPE and up to 25% of hospitalized patients are in contact precautions for MRSA/VRE.^6^ Decreasing contact precautions for these organisms may have a major impact on healthcare waste. We sought to describe the environmental and corresponding human health impact of contact precautions for MRSA/VRE in one of the largest counties in the US.

## Methods

We conducted a prospective, observational, descriptive investigation of PPE required for care of adults (≥18 years old) admitted to acute care hospitals in Los Angeles County during 2023. To quantify PPE use attributable to MRSA/VRE contact precautions, we observed care for patients: 1) in contact precautions with MRSA; 2) in contact precautions with VRE; 3) with methicillin-susceptible *Staphylococcus aureus* (MSSA) not in contact precautions, and 4) with vancomycin-susceptible *Enterococcus* (VSE) not in contact precautions.

We conducted 24 direct observations of patient care per group (MRSA, MSSA, VRE, VSE) to quantify PPE use. Observations exclusively quantified PPE use without assessing appropriateness of use. Based on a minimum expected difference of 40% less PPE use in non-MDRO groups versus MDRO groups, 24 observations per group would have >90% power to identify such a difference. Each observation lasted one hour and observations were distributed throughout the day (7AM–7PM). Observations were performed at six different hospitals representing community, academic, and safety-net facilities that used contact precautions with disposable gowns for MRSA and VRE. To balance patient characteristics between groups (MDRO vs non-MDRO), observations were matched for hospital type, care setting (intensive care unit, ward, and stepdown), and time of day. Institutional Review Board approval was obtained through the LA County Department of Public Health.

We surveyed hospitals for the average daily number of hospitalized patients in 2023 in contact precautions for MRSA and/or VRE with disposable gowns. We calculated the percentage of licensed beds in LA County acute care hospitals occupied by patients in contact precautions for MRSA/VRE using the number of licensed beds at all hospitals that responded to our survey: Percentage of licensed beds occupied by patients in MRSA/VRE contact precautions = Number of patients in contact precautions for MRSA/VRE/total number of licensed beds.

PPE use was pooled across sites to model MRSA/VRE contact precautions’ PPE use in one year:

[PPE per year (MDRO)—PPE per year (non-MDRO)] * Patients in MDRO contact precautions.

We used a process-based, environmental life cycle impact assessment (LCA) approach to evaluate the environmental and human health impact of MRSA/VRE contact precautions. The modeled inventory was based off a single, 63g^9^ nonwoven polypropylene gown disposed of as municipal solid waste in a sanitary landfill and extrapolated to represent LA County gown consumption. This inventory was mapped to the Ecoinvent v3.10 cut-off by classification database, and analyzed with ReCiPe 2016 Midpoint (H) V1.08/World (2010) in SimaPro v9.4.0.3 (PRé Sustainability, The Netherlands).

## Results

Mean gown use was higher for MRSA versus MSSA (2.33 vs. 0.17 gowns/hour, *p* < 0.01) and VRE versus VSE (2.50 vs. 0.59 gowns/hour, *p* < 0.01) (Table 1). Glove use did not vary between MRSA versus MSSA (5.08 vs 4.33 gloves/hour, *p* = 0.45) or VRE versus VSE (5.88 vs. 4.46 gloves/hour, *p* = 0.50).

**Table 1.** Average gown & glove use per hour by organism and site. Between 2 and 6 observations were conducted per hospital per organism, for a total of 96 observations among all groups (MRSA, MSSA, VRE, and VSE)

	Hospital 1	Hospital 2	Hospital 3	Hospital 4	Hospital 5	Hospital 6
MRSA Gowns per hour	2.0	3.0	1.8	1.3	2.0	4.5
MRSA Gloves per hour	4.7	6.3	3.6	3.3	5.0	9.0
MSSA Gowns per hour	0.2	0.0	0.0	1.0	0.0	0.0
MSSA Gloves per hour	4.7	6.3	2.4	5.3	1.0	4.0
VRE Gowns per hour	2.2	3.2	1.0	4.7	2.0	2.5
VRE Gloves per hour	4.3	6.7	3.8	10.0	4.0	7.0
VSE Gowns per hour	0.0	0.7	0.8	0.0	0.5	0.0
VSE Gloves per hour	4.0	4.7	4.4	6.0	4.5	3.0

Sixty-nine of the 81 hospitals (85%) in LA County responded to our survey. In aggregate, 2.9% of licensed beds in LA County acute care hospitals are occupied by patients in contact precautions with disposable gowns for MRSA or VRE. Using the assumptions of 2.16/1.91 extra gown use per hour (MRSA/VRE, respectively), 16 hours per day (excluding 8 hours of nighttime, which has less patient contact), 365.25 days/year, and 2.9% of licensed hospital beds occupied by patients in contact precautions for MRSA/VRE, approximately 7.3 million single-use disposable gowns per year are consumed specifically for MRSA/VRE contact precautions.

Single-use gowns used for MRSA/VRE contact precautions create 459,900kg plastic waste per year in LA County. Our LCA demonstrates that manufacture and disposal of these 7.3 million gowns generate 1.73 million kg CO_2_ equivalents annually. Resultant environmental damages from this level of gown consumption equate the loss of 4.07 disability-adjusted life-years (DALYs) yearly.

## Discussion

We found that gown use, but not glove use, was higher in patients in MRSA/VRE contact precautions versus patients with MSSA/VSE not in contact precautions. We estimate that MRSA/VRE contact precautions across LA County produce >500 tons of plastic waste and generate 1.73 million kg CO_2_ equivalents, with human health impacts resulting in the loss of 4.07 DALYs per year.

Excess gown use from MRSA/VRE contact precautions emits CO_2_ equivalents equal to driving 4.4 million miles in a gas-powered vehicle.^10^ Our observed DALY loss demonstrates that excess gown use adversely affects health due to the production and disposal of gowns used for MRSA/VRE contact precautions. Waste-reducing strategies such as reserving PPE use for high-contact patient care activities or increasing utilization of reusable gowns, which have lower environmental impact,^9^ should be considered for MRSA/VRE infected or colonized patients. A modest 50% decrease in gown waste from MRSA/VRE contact precautions in LA County would reduce plastic waste by over 500,000 pounds yearly.

Glove use did not differ significantly between care of patients in contact precautions and patients in standard precautions. Inappropriate glove use is common, has been linked to reduced hand hygiene rates, and contributes substantially to healthcare plastic pollution. Educational and behavioral interventions addressing glove use can improve environmental impact without compromising infection prevention.

Limitations of this study include estimates based on a limited number of hospitals in one geographic region, a small number of observations per site and unit type, and using a single type of gown and disposal approach for environmental modeling. Strengths of our study include a conservative approach to PPE modeling (i.e., not including nighttime PPE usage) and measuring gown/glove use for non-drug resistant organisms (MSSA and VSE) which provides better characterization of the PPE required specifically for MRSA/VRE contact precautions.

Our novel study isolates and quantifies environmental and human health impacts from a single, controversial infection prevention practice. We add to the body of evidence demonstrating that MRSA/VRE contact precautions may have harmful effects upon patients, healthcare operations, and costs. To prevent overall health consequences, abandoning or limiting the use of these precautions should be considered.
